# Mutations that are a common cause of Leber congenital amaurosis in northern America are rare in Southern India

**Published:** 2009-09-04

**Authors:** Periasamy Sundaresan, P. Vijayalakshmi, Stewart Thompson, Audrey C. Ko, John H. Fingert, Edwin M. Stone

**Affiliations:** 1 Department of Genetics, Dr. G. Venkataswamy Eye Research Institute, Aravind Medical Research Foundation, Madurai, Tamilnadu, India; 2 Department of Paediatric Ophthalmology, Aravind Eye Hospital, Madurai, Tamilnadu, India; 3 Howard Hughes Medical Institute, Department of Ophthalmology and Visual Sciences, University of Iowa, Iowa City, IA; 4 Department of Ophthalmology and Visual Sciences, University of Iowa, Iowa City, IA

## Abstract

**Purpose:**

To test patients from southern India for the presence of mutations that most commonly cause Leber congenital amaurosis (LCA) in northern America.

**Methods:**

A review of the literature identified 177 unique LCA causing mutations in eight different genes: aryl hydrocarbon receptor interacting protein-like 1 (*AIPL1*), crumbs homolog 1 (*CRB1*), cone-rod homeobox (*CRX*), guanylate cyclase 2D (*GUCY2D*), nephronophthisis 6 (*NPHP6*), retinol dehydrogenase 12 (*RDH12*), retinal pigment epithelium-specific protein 65 kDa (*RPE65*), and retinitis pigmentosa GTPase regulator interacting protein 1 (*RPGRIP1*). Allele-specific ligation assay and bidirectional sequencing were used to test 38 unrelated LCA patients from southern India for 104 of these mutations, which contribute to more than 30% of the LCA cases in a northern American population.

**Results:**

Only one participant was found to harbor one of the 104 mutations in the allele-specific assay (homozygous *RPE65* Tyr368His). A mutation that was not part of the assay (homozygous *RPE65* Tyr143Asp) was incidentally detected in a second patient when an equivocal signal from one allele on the assay was followed up with automated DNA sequencing.

**Conclusions:**

Mutations that contribute to 30% of the LCA cases in northern America were detected in only 2.6% of LCA cases in our cohort from southern India. There were no instances of IVS26 c.2991+1655 A>G in *NPHP6*, the most commonly detected mutation in LCA. These data suggest that LCA in India is caused primarily by a different set of mutations in the same genes associated with disease in northern America, or by mutations in other genes that have not yet been discovered. Therefore, mutation-specific assays developed for European and northern American cohorts may not be suited for testing LCA patients from India or other ethnically distinct populations.

## Introduction

Leber congenital amaurosis (LCA; OMIM 204000) is a term used to describe a heterogenous and typically autosomal recessive group of inherited retinal dystrophies characterized by: severe visual impairment at birth; normal-appearing retina; and profoundly reduced electroretinogram. Thirteen genes have been associated with LCA**:** aryl hydrocarbon receptor interacting protein-like 1 (*AIPL1)* [1], crumbs homolog 1 (*CRB1)* [2], cone-rod homeobox (*CRX)* [3], guanylate cyclase 2D (*GUCY2D*; *RETGC1*) [4], inosine monophosphate dehydrogenase 1 (*IMPDH1)* [5], Leber congenital amaurosis 5 (*LCA5)* [6], lecithin retinol acyltransferase *(LRAT)* [7], nephronophthisis 6 (*NPHP6; CEP290*) [8], retinol dehydrogenase 12 (*RDH12)* [9], retinal degeneration 3 (*RD3)* [10], retinal pigment epithelium-specific protein 65 kDa (*RPE65)* [11], and retinitis pigmentosa GTPase regulator interacting protein 1 (*RPGRIP1)* [12], tubby like protein 1 (*TULP1)* [13,14]. The frequency of disease-associated mutations among eight of these genes has recently been assessed in large populations of patients, using a range of technologies [15-18]. Many of the variations detected in these studies were only rarely observed, but nearly 100 different variations were detected in two or more unrelated LCA patients [18].

In one study, mutations in *GUCY2D*, *RPE65,* and *CRX* were detected in LCA patients from India, although at lower frequency than previously reported in cohorts from northern America [19]. In another study, mutations in *RPE65* were similarly detected in a smaller proportion of LCA patients from India than in northern America [20]. Finally, a 2006 [10] study of *RD3* identified a mutation in an Indian family, but found no examples in LCA patients from northern America. Although these studies have explored the role of some previously discovered LCA genes in causing disease in patients from India, no comprehensive investigation of multiple genes has been performed on this population. We set out to determine the frequencies of mutations associated with LCA in the Indian population by testing 38 unrelated LCA patients from India for 104 previously identified mutations in eight genes. In a 2007 study [18] of 642 northern American LCA patients, 49 of these 104 mutations cumulatively contributed to more than 30% of LCA cases.

## Methods

The study received approval from the Ethical Review Board of Aravind Eye Care System in India, and the Institutional Review Board of the University of Iowa (Iowa City, IA). Informed consent was obtained from all participants. Patients were examined at the Aravind Eye Hospital (Madurai, India), a regional facility serving a large area of southern India. As such, patients and controls recruited for this study represent an unselected sampling of the South Indian population. A total of 41 unrelated individuals were enrolled in this study as probands after a diagnosis of LCA. Probands were from 6 months to 12 years of age at enrollment (mean 5.0 years SD±3.3), 24 female and 17 male, and in good health. Diagnosis of LCA was based on the following criteria: 1) severely reduced vision in both eyes that was recognized within two years of birth; 2) relatively normal appearance of the retina; 3) profoundly reduced or nonrecordable ISCEV electroretinogram; and 4) absence of symptoms that would suggest another disease. When possible, relatives were also assessed and included in the study for assessment of inheritance phase. A total of 84 family members were enrolled, this included: 74 parents (0 affected; 33 male, 41 female; mean age 32.2 years SD±7.5), and 12 siblings (2 affected, 1 female aged 2 and 1 male aged 7; 10 unaffected, 6 male, and 4 female; mean age 10.7 years SD±4.0). Although each proband was unrelated, consanguinity was identified from histories in the families of 34 of 41 probands (83%). Additionally, 25 unrelated healthy individuals were used as control study subjects.

### DNA preparation

Peripheral blood (approximately 10 ml) was collected using vacutainer EDTA (EDTA) tubes (Becton-Dickinson, Franklin Lakes, NJ), and DNA was extracted by salt precipitation [21]. Blood samples were obtained from 41 unrelated affected individuals with LCA. However, the concentration of the DNA obtained from three of these participants was inadequate for the allele-specific ligation assay. Consequently, only 38 individuals were studied with both the allele-specific ligation assay and bidirectional sequencing of *NPHP6*, as will be described.

### Allele-specific ligation assay

LCA-associated mutations in *GUCY2D*, *RPE65*, *CRB1*, *AIPL1*, *CRX*, *RDH12*, and *RPGRIP1* have been well characterized in northern American populations [18]. A total of 177 previously reported mutations in these genes were identified in a review of the literature. A multiplexed allele-specific assay was designed to detect 138 of these mutations using the SNPlex platform (Applied Biosystems, Foster City, CA). Four of the 138 probes were subsequently found to be benign polymorphisms [18], or evidence for disease causing status was inconclusive (RPGRIP1 ARG812GLN). These probes were therefore excluded from analysis. Of the remaining 134 plausible disease-causing alleles, technical limitations in the probe sets meant the assay was unable to reliably detect either allele for 31 single nucleotide polymorphisms (SNPs). Consequently, genotypic data from 103 SNPs were included in our analysis (Table 1).

**Table 1:** Genotypes for deliberately assessed alleles.

**Gene**	**Mutation**	**NL**	**Mut**		**Gene**	**Mutation**	**NL**	**Mut**		**Gene**	**Mutation**	**NL**	**Mut**	
**Het**	**Hom**	**Het**	**Hom**	**Het**	**Hom**
*AIPL1*	VAL71PHE	38	0	0	*GUCY2D*	MET1ILE	38	0	0	*RPE65*	GLY40SER	38	0	0
	MET79THR	37	0	0		LEU41PHE	38	0	0		ARG44GLN	38	0	0
	TRP88STOP	37	0	0		TYR173ins6tACGCCC	38	0	0		GLY46del1G	38	0	0
	CYS89ARG	37	0	0		ARG313CYS	38	0	0		ARG91GLN	38	0	0
	ALA197PRO	38	0	0		LEU325PRO	37	0	0		ARG91TRP	38	0	0
	LYS242del3AAG	38	0	0		SER448STOP	38	0	0		GLU102STOP	38	0	0
	TRP278STOP	38	0	0		ARG540CYS	38	0	0		ARG124STOP	38	0	0
	LEU293PRO	38	0	0		ARG660STOP	38	0	0		ALA132THR	37	0	0
	ARG302LEU	38	0	0		TYR746CYS	38	0	0		THR162PRO	37	0	0
	IVS3 1G>A	38	0	0		GLU750STOP	34	0	0		ASN205del2aaCA	38	0	0
*CRB1*	PHE144VAL	38	0	0		ARG768TRP	38	0	0		ARG234STOP	38	0	0
	THR289MET	38	0	0		THR839ALA	38	0	0		TYR239ASP	37	0	0
	CYS383TYR	38	0	0		LEU954PRO	38	0	0		VAL287PHE	38	0	0
	CYS681TYR	38	0	0		SER981del1G	38	0	0		TYR318ASN	38	0	0
	GLU710GLN	38	0	0		CYS984TYR	38	0	0		CYS330TYR	38	0	0
	MET741THR	38	0	0		MET1009LEU	38	0	0		LEU341SER	36	0	0
	PRO748del3cCAT	38	0	0		HIS1019PRO	38	0	0		ALA360PRO	38	0	0
	ARG764CYS	38	0	0		ARG1029SER	38	0	0		TYR368HIS	37	0	1
	LYS801STOP	38	0	0		GLN1036STOP	38	0	0		ALA393GLU	38	0	0
	GLY827STOP	38	0	0		IVS9–2T>A	38	0	0		GLU417GLN	38	0	0
	ILE852THR	38	0	0		IVS16–4A>T	33	0	0		TRP460CYS	38	0	0
	ASN871ins1aaT	34	0	0	*NPHP6*	IVS26 c.2991+1655	38	0	0		GLU462STOP	38	0	0
	CYS896STOP	38	0	0	*RDH12*	THR49MET	38	0	0		VAL473ASP	38	0	0
	SER1025ILE	38	0	0		LEU99ILE	38	0	0		GLY528VAL	38	0	0
	ILE1100ARG	38	0	0		GLY127STOP	38	0	0	*RPGRIP1*	ASP248HIS	38	0	0
	LEU1107ARG	38	0	0		HIS151ASP	38	0	0		SER502ins4tcTGTC	38	0	0
	LEU1107PRO	35	0	0		SER175PRO	37	0	0		ARG580GLY	36	0	0
	TRP1293STOP	38	0	0		TYR194STOP	35	0	0		GLY746GLU	38	0	0
	ASN1317HIS	38	0	0		ALA206ASP	38	0	0		LEU856ins2cTT	38	0	0
	CYS1321GLY	35	0	0		TYR226CYS	38	0	0		ASP877GLY	38	0	0
	GLU1330del1G	37	0	0		PRO230ALA	38	0	0		GLU1279del3GAG	32	0	0
	IVS10–1G>T	38	0	0		ALA269del5CCCTG	38	0	0		IVS8–3A>G	37	0	0
*CRX*	GLU173del1G	38	0	0		IVS5–1G>A	38	0	0		IVS15–1G>A	38	0	0
	VAL180del1G	38	0	0							IVS16–1G>A	38	0	0
	TYR191del1T	38	0	0										
	TYR195STOP	35	0	0										
	GLY217del1G	35	0	0										

The presence or absence of LCA-causing alleles as assessed by SNPlex allele specific ligation assay and bidirectional sequencing (NPHP6 IVS26 c.2991+1655 only) are shown by gene. For each allele assessed, the number of proband samples identified as homozygous normal (NL), heterozygous for a disease causing allele (Mut - Het) or homozygous for a disease causing allele (Mut - Homo) are shown. Any discrepancy between the sum of these numbers and the 38 samples assayed is due to failed allele calls.

DNA sample preparation, allele-specific ligation, and post-ligation amplification were performed in 96 well plates according to the manufacturer’s instructions (SNPlex, Applied Biosystems). PCR products were analyzed with a 3730 DNA sequencer and GeneMapper software (Applied Biosystems). Allele status was initially assigned using custom software developed at the University of Iowa, and allele assignments were then confirmed manually. In some cases, genotypes could not be reliably assigned because of low probe signal strength (below 500 units for both normal and mutant alleles). In total 51 of 3,914 genotypes (1.3%) were excluded for this reason.

Positive results from the allele-specific assay were verified by bidirectional sequencing of an amplimer spanning the mutation in question. Confirmed mutations were assessed in relatives of the probands to establish phase. Approximately 150 ng of each patient's DNA was used as template in a 30.0 µl PCR containing the following: 3.0 µl 10X buffer (100 mM Tris-HCl pH 8.3, 500 mM KCl, 15 mM MgCl_2_), 9 mM of each dCTP, dATP, dGTP, and dTTP, 9 pmole of each primer (Integrated DNA Technologies, Coralville, IA), and 0.9 units of DNA polymerase (Biolase, Irvine, CA). Samples were denatured for 5 min at 94 °C and incubated for 35 cycles under the following conditions: 94 °C for 30 s, 57 °C for 30 s, 72 °C for 30 s in an MJ Research DNA thermocycler (BioRad, Waltham, MA). PCR products were purified with the QIAquick PCR purification kit (Qiagen, Valencia, CA). Sequencing was by dye-termination chemistry on an ABI 3730 DNA sequencer (Applied Biosystems), with subsequent sequence analysis using Sequencher (Gene Codes Corporation, Ann Arbor, MI).

### *NPHP6* IVS26 c.2991+1655 mutation screening

In the northern American population, the single most common LCA-associated mutation is a deletion within intron 26 of *NPHP6* (IVS26 c.2991+1655). Therefore a screen of this mutation was conducted in parallel to the SNPlex assay. The presence or absence of the c.2991+1655 mutation in intron 26 of *NPHP6* was assessed by bidirectional sequencing of genomic DNA from each unrelated affected individual. Only those also studied through the SNPlex assay (n=38) were included in this report. Reaction conditions and sequence analysis were as described in the previous section, except annealing conditions were as follows: 30 s, with 65 °C for the first cycle, reducing in temperature increments over the next nine cycles to 60 °C, followed by 25 cycles at 60 °C.

## Results

The presence or absence of 103 LCA-causing mutations was successfully assayed with an allele-specific ligation assay applied to 38 patients with LCA from southern India. Genotype was determined in 3,755 of 3,914 tests, giving a sensitivity of 95.9% for these alleles (Table 1; the 104^th^ allele was assayed only by bidirectional sequencing, please see paragraph referring to *NPHP6* below). Of these disease-causing mutations previously found in the northern American population, only one example was identified in the cohort from South India.

**Figure 1:**
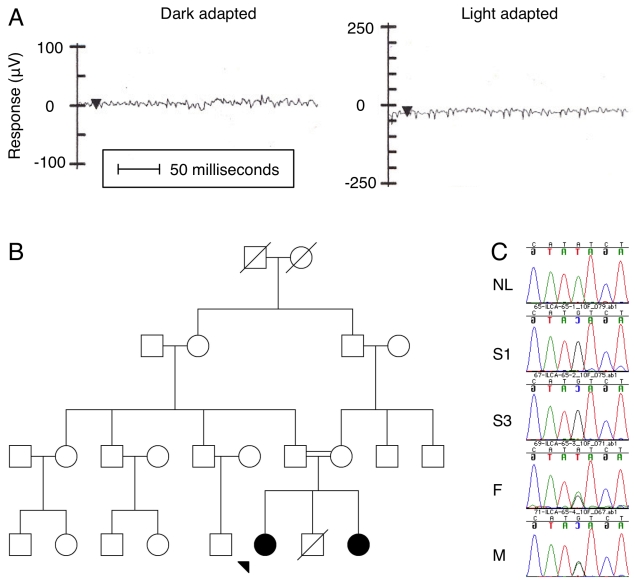
Clinical and molecular data for patient ILCA-65–1 and family. The proband first presented for ophthalmic examination at 3 years of age. **A**: There were no recordable responses to light in the electroretinogram. Examples shown are from the left eye for a dark-adapted combined response and a light-adapted photopic response. Arrowhead points to the timing of the 10-ms bright light pulse. **B**: The family tree shows the proband (filled circle with arrow) and a sibling as clinically affected and both parents as unaffected. **C**: Bidirectional sequencing showed that the *RPE65* Tyr368His TAT>CAT mutation was homozygous in the affected proband (S1) and affected sibling (S3), and was heterozygous in the mother (M) and father (F). Reverse strand sequence around *RPE65* Tyr368 (caNatct) is shown against an ethnic unrelated and unaffected control normal (NL).

Patient ILCA-65–1 was homozygous for the mutation (Tyr368His TAT>CAT) in *RPE65* (Figure 1). This patient presented at the Aravind Eye Institute at 3 years of age with a history of poor vision. On examination, visual acuity was 20/80 (6/24) in the right eye and 20/200 (6/60) in the left eye. Both dark-adapted scotopic and light-adapted photopic electroretinograms were nonrecordable. At age 9, pigmentary changes were observed in the midperiphery of the the child’s fundus. Unaffected parents and an affected sibling were available for study. The affected sibling was examined at 2 years of age and had visual acuities of 20/120 (6/36) in the right eye and 20/160 (6/48) in the left eye, extinguished electroretinogram responses, and pigmentary retinopathy. The presence of this mutation was confirmed with bidirectional DNA sequencing and the phase established in available samples from relatives. Both unaffected parents were found to carry a heterozygous Tyr368His mutation, while the affected sibling was homozygous for the Tyr368His mutation.

**Figure 2:**
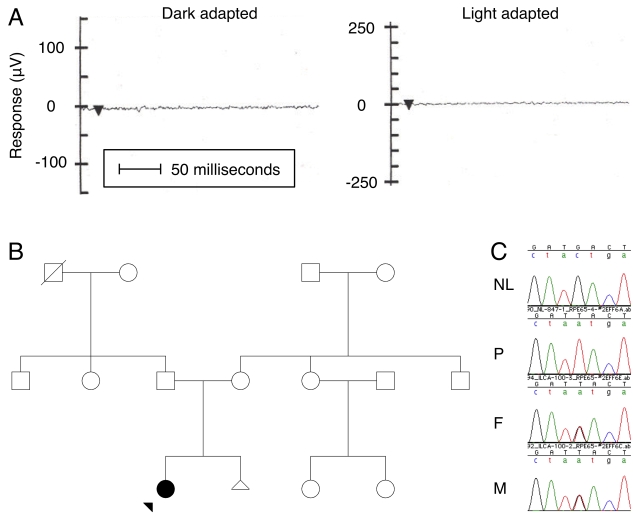
Clinical and molecular data for patient ILCA-100–1 and family. The proband first presented for ophthalmic examination at 4 months of age. When the proband was 3 years old **(A),** there were no recordable responses to light in the electroretinogram. Examples shown are from the left eye for a dark-adapted combined response and a light-adapted photopic response. Arrowhead marks the timing of the 10-ms bright light pulse. **B**: The family tree shows the proband (filled circle with arrow) as clinically affected, and both parents are unaffected. **C**: Bidirectional sequencing showed that the *RPE65* Tyr143Asp TAC>GAC mutation was homozygous in the affected proband (P) and was heterozygous in the mother (M) and father (F). Reverse strand sequence around *RPE65* Tyr143 (atNacta) is shown against an ethnic unrelated and unaffected control normal (NL).

In one instance, a mutation detected by the allele-specific assay was not confirmed by subsequent DNA sequencing. A Glu102STOP mutation in the *RPE65* gene of one patient (ILCA-100–1) was suggested by the allele-specific assay. However, DNA sequencing showed this to be a false positive, and identified a different *RPE65* mutation that was not included on our allele-specific assay (a homozygous Tyr143Asp TAC>GAC; Figure 2). Patient ILCA-100–1 was first presented at the Aravind Eye Institute at 4 months of age with the mother’s complaint that the infant was not fixating on the her face. The patient was full term at birth, with normal delivery and no remarkable antenatal or perinatal problems. At 3 years of age, the patient’s examination was consistent with LCA: severe visual impairment with nonrecordable electroretinogram in both eyes, normal anterior segment, and abnormal pigmentation in all four quadrants of the fundus with mild temporal pallor of the discs in both eyes. At age 4, on a recent follow-up, the subject was found to have a visual acuity of 20/120 (6/36) with both eyes open, and has achieved otherwise normal developmental milestones. Again, family history identified consanguinity, and both unaffected parents were found to be heterozygous for the Tyr143Asp mutation.

The same cohort of 38 LCA patients were tested for the c.2991+1655 A>G mutation in intron 26 of *NPHP6* with bidirectional DNA sequencing. No instances of this mutation were detected.

## Discussion

LCA is a heterogeneous condition that is responsible for severe vision loss at birth. In the past 10 years great progress has been made in identifying the genes that are responsible for this condition. Mutations in the eight genes we studied in the South Indian population account for 64% of LCA in the United States [18].

In the current study, we explored the role of previously discovered LCA mutations in a cohort of patients from southern India by testing for the presence of 104 previously reported mutations in eight LCA genes. While this particular set of mutations has been associated with roughly 30% of LCA cases in prior studies of patients from northern America, only one proband in the cohort from southern India (ILCA-65–1) was found to carry one of these 104 mutations previously associated with LCA.

Our SNPlex assay incidentally detected a plausible disease-causing mutation in the *RPE65* gene that was not one of the 103 alleles included in the original assay design. A homozygous Tyr143Asp mutation was identified in subject ILCA-100–1. Segregation of the mutation with disease in the family supports the Tyr143Asp mutation as the cause of disease in this subject. Although we had not previously observed Tyr143Asp in LCA, we have observed Tyr143Asp as one of the disease-causing mutations in a patient categorized as having early onset retinitis pigmentosa. The relatively good vision for an LCA patient and early pigmentary changes of ILCA-100–1 illustrate the potential for overlap in these clinical categories when the cause of disease can be traced to mutations in the same gene.

The lower prevalence of mutations in the cohort of patients from India is likely due in part to the high rate of novel mutations that are detected in LCA genes. In a prior study, 77% of disease-causing variations that were detected were only observed once [18]. Consequently, it is plausible that as for patient ILCA-100–1, many patients in our study have novel mutations in *GUCY2D*, *RPE65*, *CRB1*, *AIPL1*, *CRX*, *RDH12*, *RPGRIP*, or *NPHP6* that were not included in the mutation-specific assay. Alternatively, it is possible that a large proportion of LCA cases from India are caused by mutations in novel or other known LCA genes (*LCA5* or *RD3*) [10,22]. Thus, our Indian cohort of patients may be a useful resource for identifying additional novel LCA genes or new mutations in known LCA genes. Furthermore, our research suggests that mutation-specific assays that are designed from studies of LCA patients from one ethnic population are not the most efficient approach for studying patients from different populations and ethnic backgrounds.

The value of a detailed molecular screen of LCA patients from the Indian population lies in genetic counseling, improved diagnosis and prognosis, the support this gives to gene discovery efforts, and, more recently, in identifying patients for treatment. In both families with a molecular diagnosis from this study, the mutation was in *RPE65*, so the recent success of gene therapy for *RPE65* presents a very real hope of treatment in the future that will improve the vision of these patients [23-25].
